# Supplementary material for: Arms races between selfish genetic elements and their host defence in termites
Source: Nat Commun. 2026 Feb 13;17:1702. doi: 10.1038/s41467-026-69550-6 (PMC12909921; doi:10.1038/s41467-026-69550-6)
Supplement: Supplementary file 1 — Supplementary Infomation [file 41467_2026_69550_MOESM1_ESM.pdf]

Supplementary Material

Arms races between selfish genetic elements and their host defence in termites

Bitao Qiu [*], Daniel Elsner, Judith Korb [*]

Supplementary Table 1. Sample information and statistics for the genome assemblies

Assembly	*Mastotermes darwiniesis*	*Zootermopsis nevadensis*	*Cryptotermes secundus*	*Reticulitermes grassei*	*Trinervitermes geminatus* *	*Odontotermes sp.2*	*Macrotermes bellicosus (Q)*	*Macrotermes bellicosus (S)*
Caste	Worker	Worker	Worker	Queen	Worker	Queen	Queen	Minor soldier
Sample age #	1-2 years	1-2 years	3-4 years	> 3 years	Few months	4 years	> 7 years	Few months
Sex	Unknown	Unknown	Unknown	Female	Unknown	Female	Female	Female
Collection site	Darwin, Australia	California, the United States	Channel Island, Australia	Île d'Oléron, France	Lamto, Côte d'Ivoire	Comoé National Park, Côte d'Ivoire	Comoé National Park, Côte d'Ivoire	Pendjari National Park, Benin
Library	HiFi standard	HiFi standard	HiFi low input	HiFi standard	HiFi low input	HiFi low input	HiFi standard	HiFi low input
Tissue	Head	Head	Gut-removed whole bodies	Gut-removed whole bodies	Gut-removed whole bodies	Fatbody	Fatbody	Gut-removed whole bodies
# contigs	2,206	321	3,582	98	3,206	1234	364	6,173
Assembly length (bp)	1,345,564,086	576,263,831	1,304,526,754	996,825,901	1,453,058,254	1,465,002,929	1,340,725,563	1,428,941,907
GC (%)	39.53	38.48	41.18	39.49	40.36	39.84	40.26	40.31
Largest contig (bp)	14,453,904	27,517,609	7,090,375	72,273,710	6,339,778	22,652,318	50,731,358	2,705,196
N50 (bp)	1,569,491	18,733,084	1,070,193	44,444,803	809,495	6,388,291	15,380,714	528,038
L50	212	13	353	10	520	73	28	813
**PacBio data statistics**								
Total reads	1,870,375	2,151,234	2,483,158	2,328,724	1,994,195	4,418,500	1,773,689	3,613,653
Raw base (Gb)	17.4	29.9	17.7	27.6	19.2	39.8	29.6	18.2
Mean read length (bp)	9,303	13,474	7,139	11,836	9,598	9,010	16,689	5,028
Read coverage	22	66	18	30	16	33	27	16
**GenomeScope results** (k-mer = 21)								
Est. haploid genome length (bp)	1,317,818,343	561,384,891	1,142,543,631	950,124,871	1,226,968,067	1,281,361,698	1,273,604,650	1,282,772,194
Est. heterozygosity (%)	0.22	0.45	5.27	0.23	1.2	0.79	0.35	0.39
Est. repeat length (bp)	547,115,452	173,947,255	591,445,906	289,378,874	858,067,778	526,497,403	499,855,941	502,589,585
**BUSCO results**								
Completeness	1364 (99.8%)	1367 (100.0%)	1364 (99.8%)	1364 (99.8%)	Pre: 1357 (99.3%)<br>Post: 1351 (98.8%)	1363 (99.8%)	1365 (99.9%)	1363 (99.8%)
Single-copy	1293 (94.6%)	1353 (99.0%)	1206 (88.2%)	1348 (98.6%)	Pre: 1161 (84.9%)<br>Post: 1315 (96.2%)	1339 (98.0%)	1316 (96.3%)	1231 (90.1%)
Duplicated	71 (5.2%)	14 (1%)	158 (11.6%)	16 (1.2%)	Pre: 196 (14.3%)<br>Post: 36 (2.6%)	24 (1.8%)	49 (3.6%)	132 (9.7%)
Fragmented	1 (0.1%)	0 (0%)	3 (0.2%)	2 (0.1%)	Pre: 7 (0.5%)<br>Post: 7 (0.5%)	3 (0.2%)	1 (0.1%)	3 (0.2%)
Missing	2 (0.2%)	0 (0%)	0 (0%)	1 (0.1%)	Pre: 3 (0.2%)<br>Post: 9 (0.7%)	1 (0.1%)	1 (0.1%)	1 (0.1%)
**Merqury results** (k-mer = 21)								
K-mer completeness	98.6 %	95.6 %	91.2 %	97.3 %	Pre: 96.1 %<br>Post: 93.9 %	93.2 %	97.2 %	96.8 %
Assembly error rates	$8x10^{-7}$	$2x10^{-7}$	$3x10^{-6}$	$1x10^{-7}$	Pre: $5x10^{-6}$<br>Post: $3x10^{-6}$	$1x10^{-6}$	$9x10^{-7}$	$2x10^{-6}$
QV	61	66	55	69	Pre: 53<br>Post: 57	60	60	57

[*] For *Trinervitermes geminatus*, the genome assembly has been processed with *purge_dups* to remove potential haplotypic duplications. BUSCO and Merqury results are provided for both the pre- and post-purging assemblies.

[#] Although there was considerable variation in chronological age among the individuals used, they were of intermediate age relative to their species and caste identity.

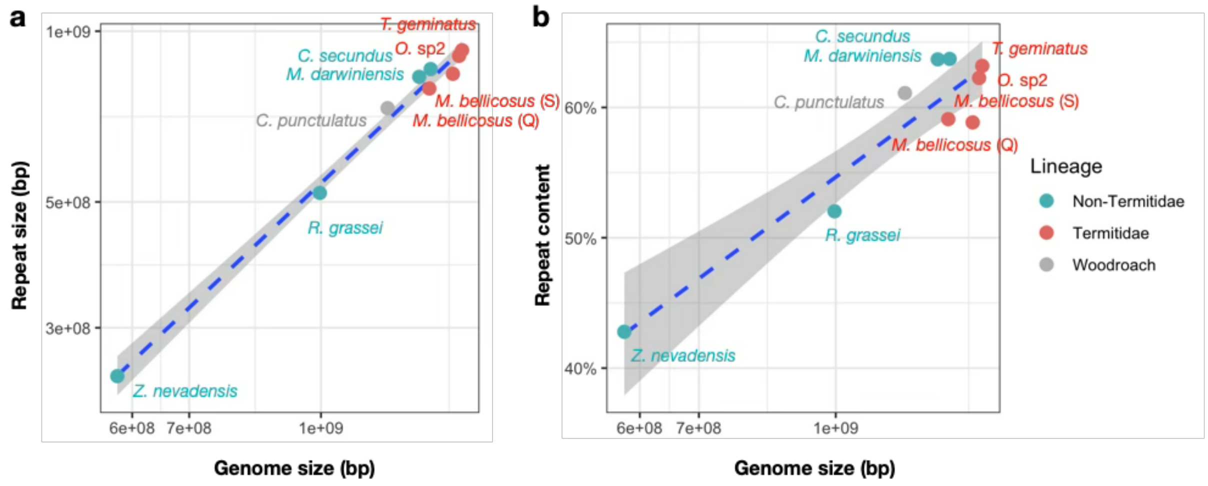

Supplementary Figure 1. Genome size of termites and the woodroach plotted against (**a**) genomic repeat size and (**b**) the proportion of repeat content (percentage of genome size). Both genomic repeat size and repeat content proportion show significant positive correlations with genome size (Pearson's correlation tests: $n_{\text{species}} = 8$, both $p < 1\times10^{-3}$).

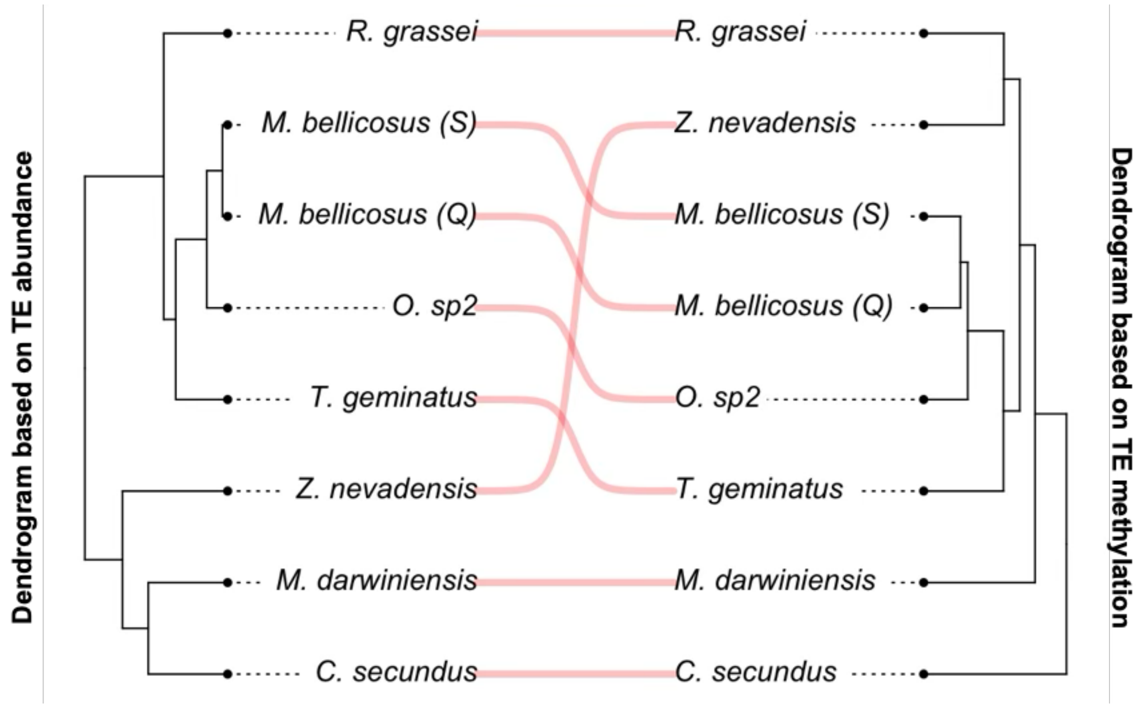

Supplementary Figure 2. Dendrogram based on TE superfamily abundance ($n_{\text{superfamily}} = 71$) (left) and on TE superfamily methylation ($n_{\text{superfamily}} = 69$) (right) across the termite phylogeny. TE abundance and TE methylation show a strong co-phylogenetic pattern (permutation test: $n_{\text{permutations}} = 100$, $p < 0.01$).

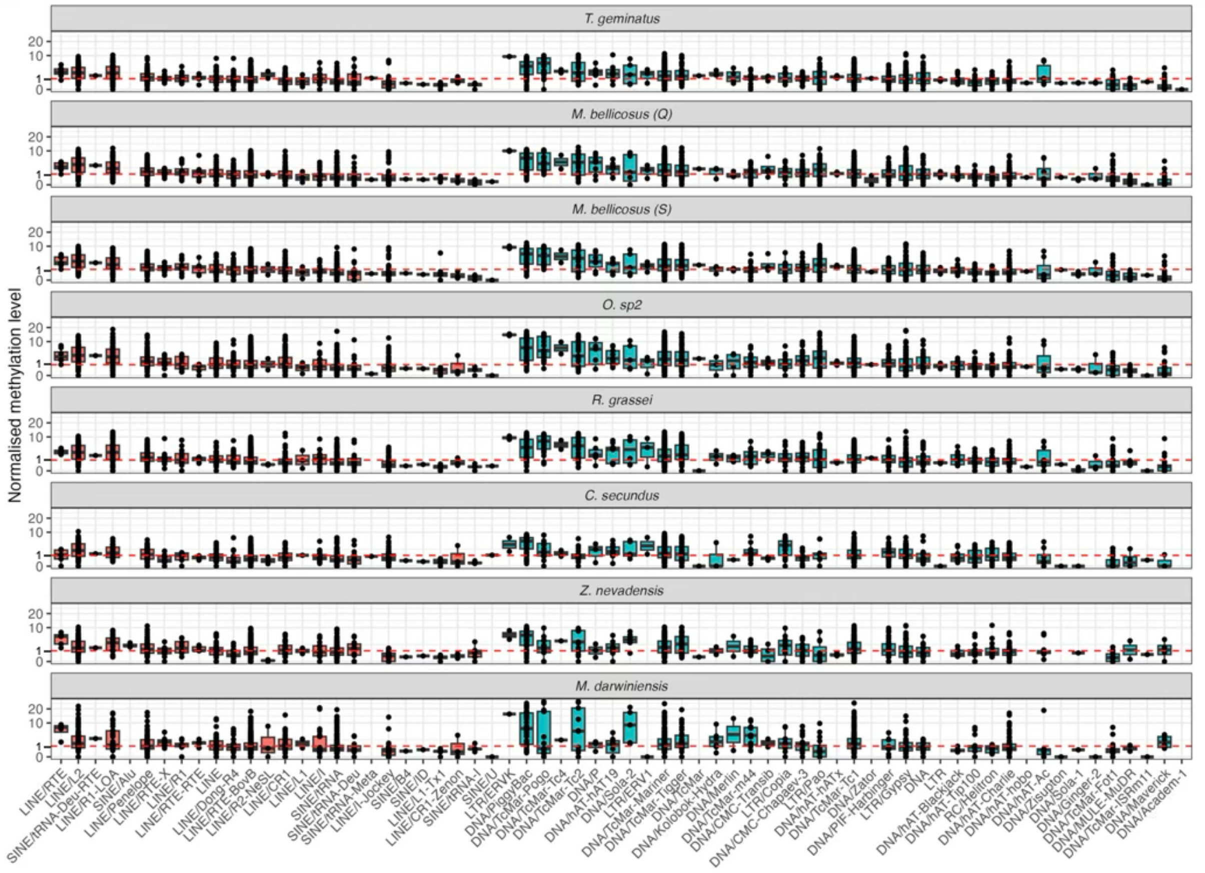

Supplementary Figure 3. Boxplots of normalized CpG methylation levels of TE superfamilies for different termite species. TE superfamilies are sorted by TE classes (red for class I TEs and blue for class II TEs) and the mean of the normalized methylation level of the corresponding TE families (points). The normalised methylation level was calculated as the ratio of the percentage of methylated CpG sites in a TE family relative to that of the genome. It ranges from 0 (devoid of methylated CpG sites), 1 (equal to the genomic background; red dashed line), to > 1 (higher than the genomic background). Within each TE superfamily, TE families showed high variation of methylation levels.

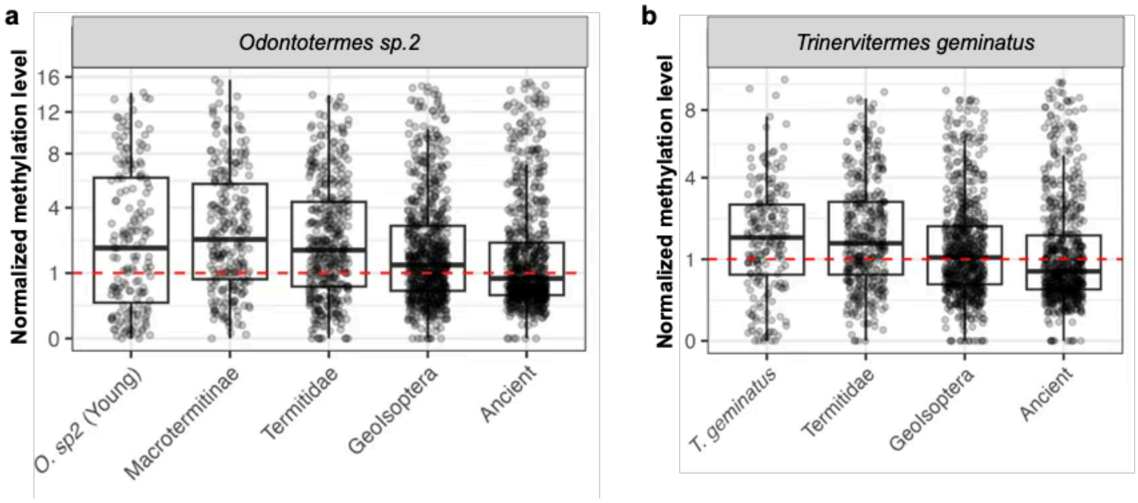

Supplementary Figure 4. Boxplots of normalized TE methylation levels across different TE age groups in (**a**) the *Odontotermes* sp.2 and (**b**) the *Trinervitermes geminatus* genome. Red dashed lines represent genomic background.

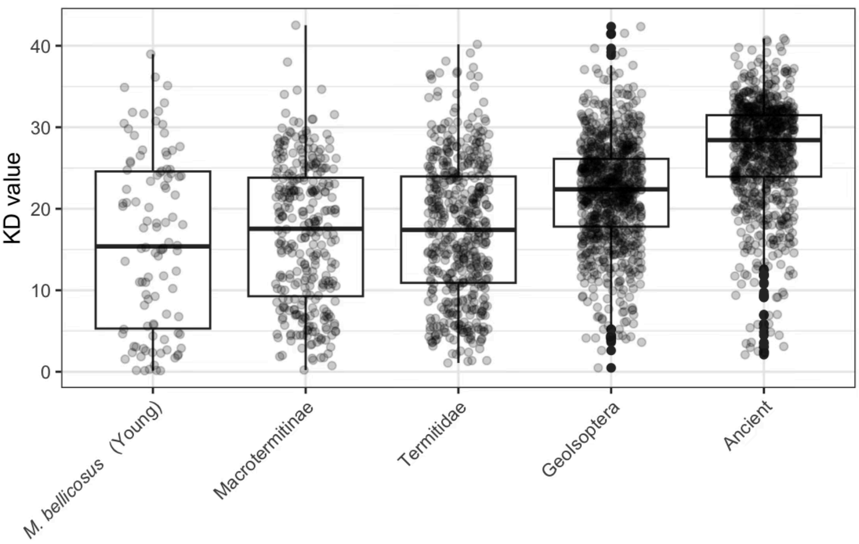

Supplementary Figure 5. Boxplots of KD values of TE families across different TE age groups. The mean KD values of ancient TE families were significantly higher than those of younger families (two-sided t-test: $n_{\text{ancient}} = 844$, $n_{\text{young}} = 103$, $p < 1\text{e-}5$), as expected since most ancient TE families are TE remnants.

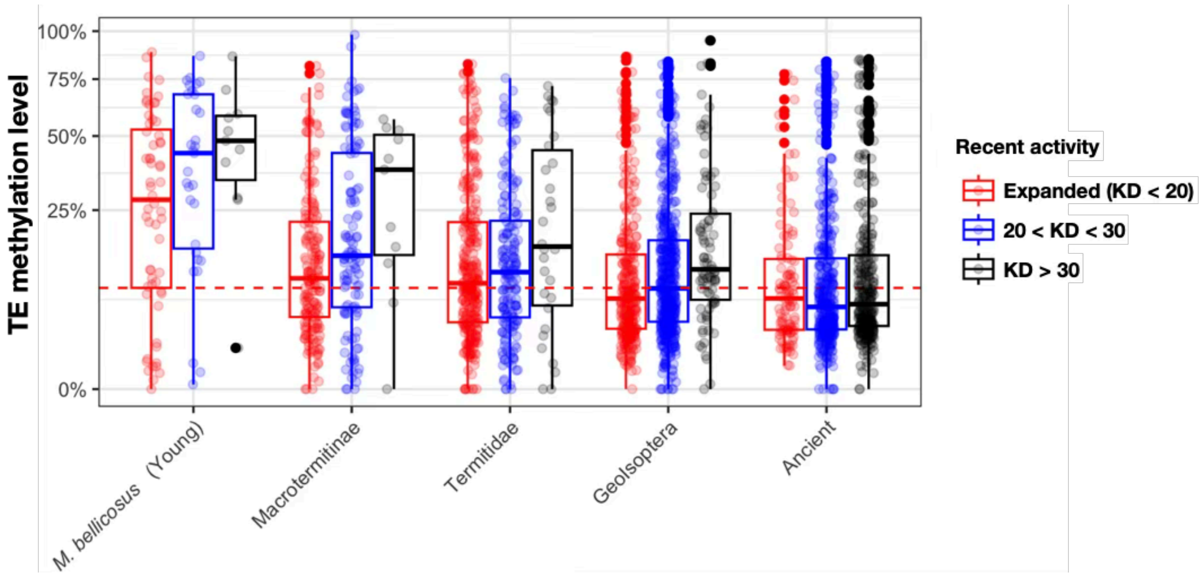

Supplementary Figure 6. Boxplots of TE methylation levels across different TE age groups. Within each age group, TE families are stratified into recently expanded (Kimura's distance, KD < 20; red) and more diverged (indication of non-active) (KD > 20; blue and black) categories. Overall, TE methylation levels declined with increasing TE age. However, more diverged TE families exhibited higher methylation levels than recently expanded families in all age groups, except for the most ancient TEs.

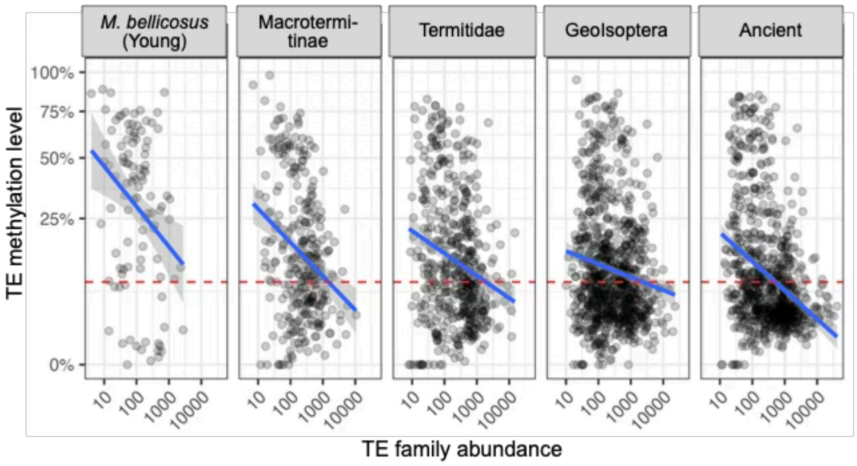

Supplementary Figure 7. Association between TE family abundance and TE methylation levels in the *M. bellicosus* genome, plotted separately for each TE age group. Each age group was fitted with a generalized regression model (blue line) and a 95% confidence band. The red dashed line represents the genomic methylation level (8%) for *M. bellicosus*.

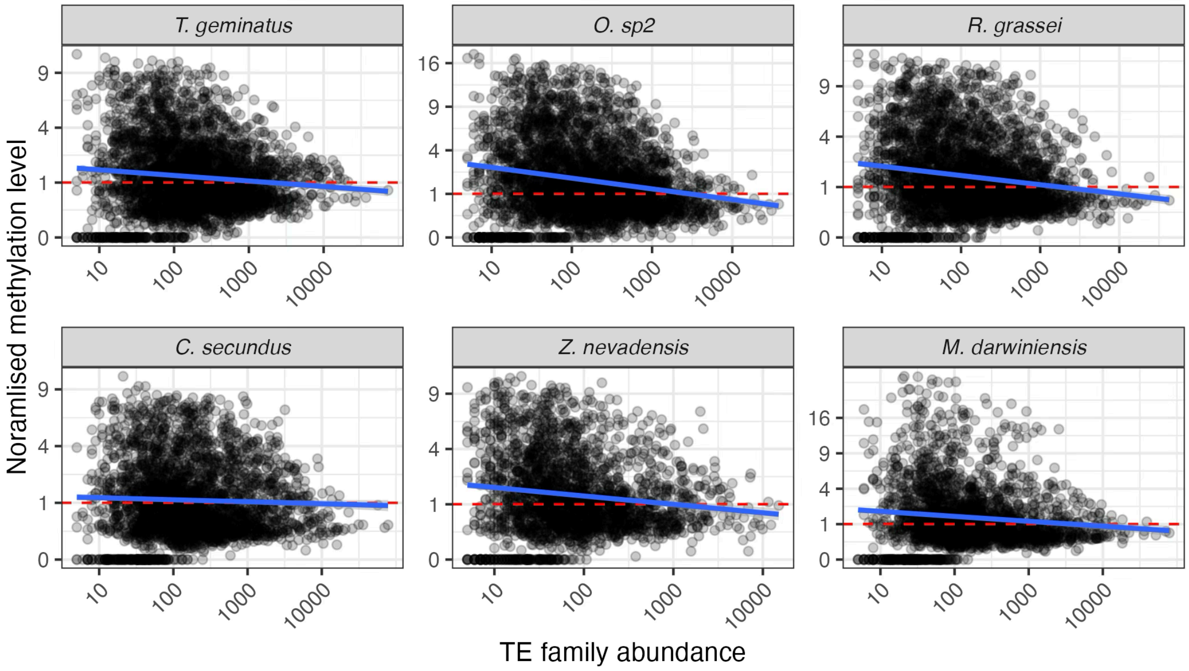

Supplementary Figure 8. Association between the normalised methylation levels and TE family abundance, plotted separately for each termite genome. TE methylation and abundance were negatively correlated in all species (Spearman's correlation test: all $p < 1e\text{-}5$, except for *C. secundus*). Each species was fitted with a generalized regression model (blue line) and a 95% confidence band. Red dashed lines represent the genomic backgrounds.

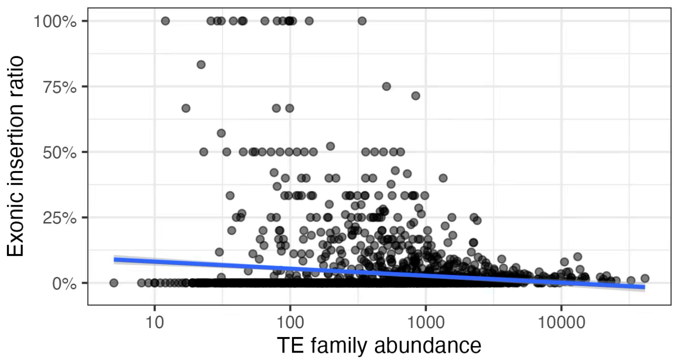

Supplementary Figure 9. Association between TE family abundance and TE-associated structural variants (SVs) exonic insertion ratios in *M. bellicosus* genomes. The exonic insertion ratio of a TE family was calculated as the percentage of exonic insertions in relation to the total number of insertions. The blue line represents the regression line and a 95% confidence band. While the majority of TE families had zero exonic insertions, TE families with high abundances were with fewer exonic insertions.

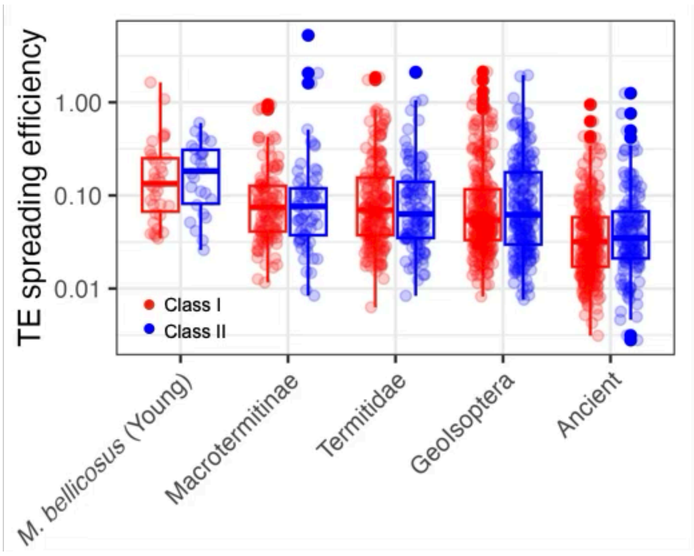

Supplementary Figure 10. Boxplots of the TE spreading efficiency across different TE ages in the two TE classes (red for class I TEs and blue for class II TEs). TE spreading efficiency was calculated as the number TE-associated SVs normalised by TE family abundance in the *M. bellicosus* genome.

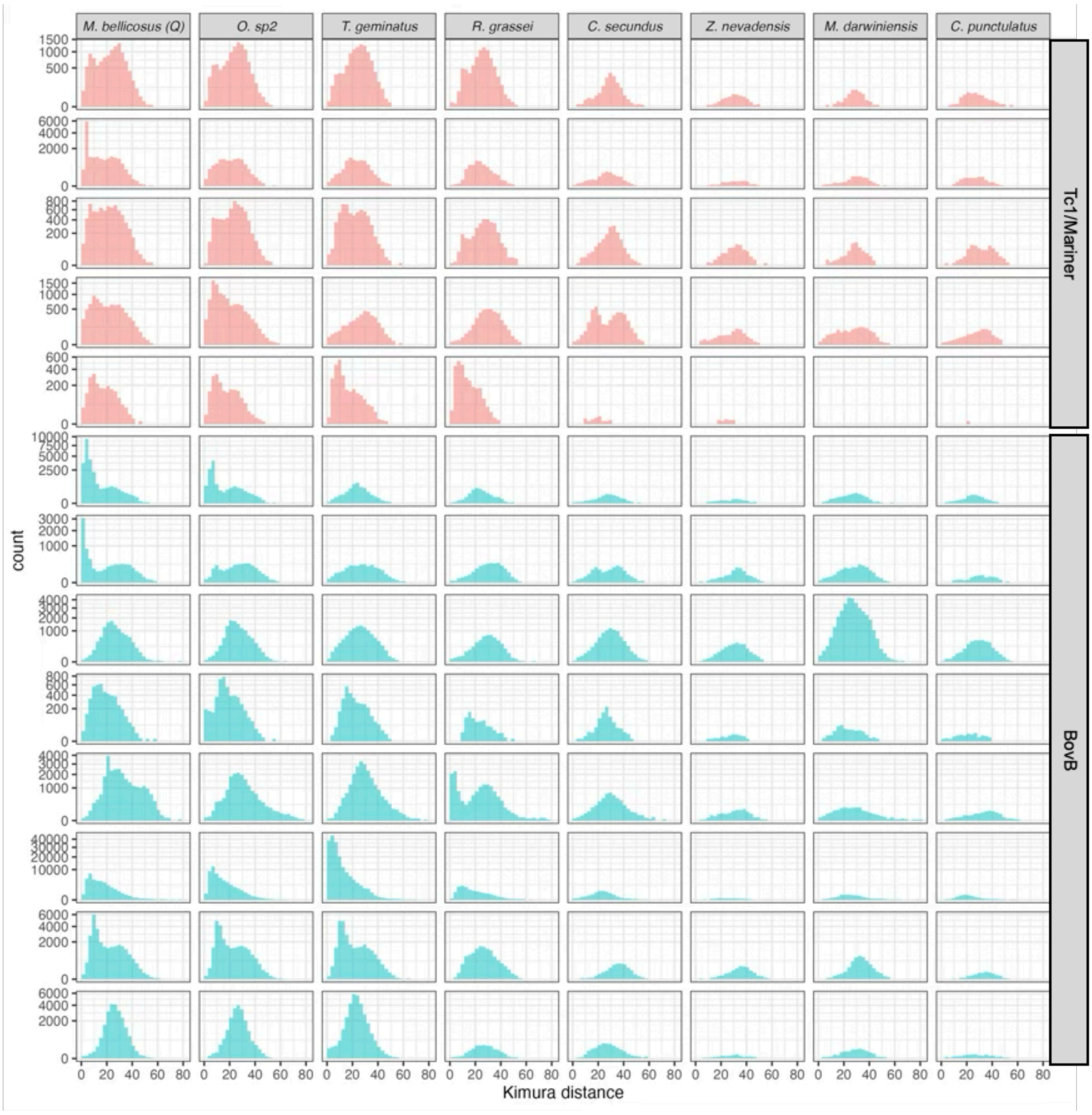

Supplementary Figure 11. Histograms of Kimura distances (KD) for the top active Tc1/Mariner (red) and BovB (blue) ancient TE families, plotted separately for each TE family (row) and each species (column). A low KD value indicates recent TE expansion/ invasion, while a high KD value suggests TE remnants that have been inactive for a long time. A combination of both (i.e., a bimodal distribution) suggests that the TE family includes both copies of inactive TE remnants and copies of recently expanded TEs.

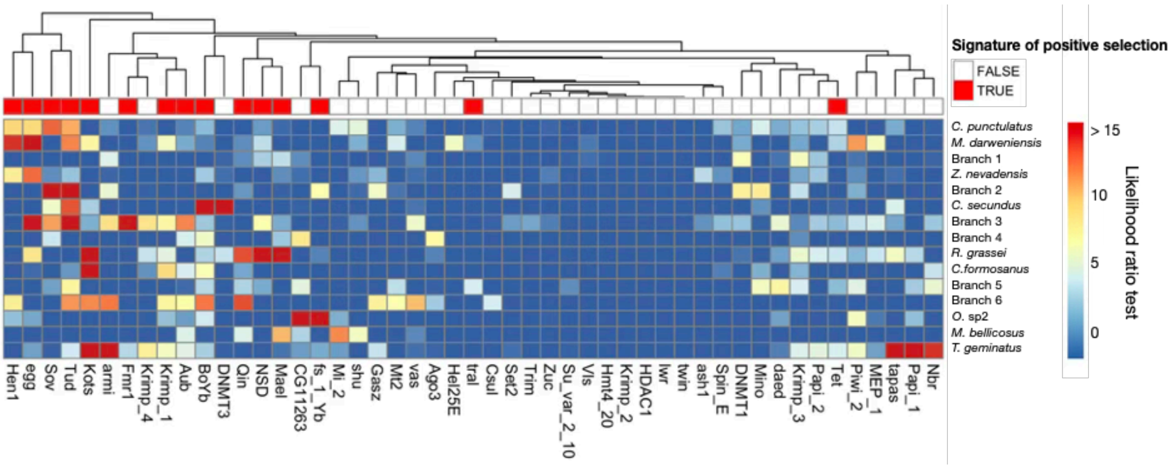

Supplementary Figure 12. Heatmap of the signatures of positive selection in TE defense genes in termites and the woodroach. Signatures of positive selection were quantified by likelihood ratio test statistics, ranging from 0 (blue) to > 15 (red). A higher value indicates stronger evidence that the gene has experienced positive selection in the target species or branch. The bar at the top of the heatmap illustrates the overall evidence for positive selection based on all species, with red indicating strong evidence ($p < 0.05$) (multiple testing correction with false discovery rate). To the right of the heatmap are the names of the tested species and branches. Branch 1, 2, 3, 4, 5 and 6 are the branch leading to the last common ancestor (BLCA) of Zootermopsis and the Icoisoptera, the BLCA of Icoisoptera, the BLCA of Geoisoptera, the BLCA of Heterotermitidae, the BLCA of Termitidae, and the BLCA of Macrotermitinae, respectively. See **Supplementary Data 3** for the full list of genes.

Supplementary Note 1. Caste effect on TE abundance and TE CpG methylation

We also looked at the caste difference in TE abundance and TE CpG methylation. Compared to the other termite genomes, the soldier and the queen genome assemblies of *M. bellicosus* were highly similar in their TE abundance and TE methylation at the TE family level (Spearman's $r = 0.99$ and 0.88, respectively). However, the queen genome had a significantly lower TE abundance and a significantly higher TE methylation level than the soldier genome (two-sided paired Wilcoxon signed-rank test: both $p < 1e\text{-}5$). As *M. bellicosus* queens have a high expression level for TE suppressing genes regardless of age [4], the TE abundance difference between the queen and the soldier genome might be associated with the lower TE activities during queen ageing.

Supplementary Note 2. Validation of CpG 5mC identification

To validate the CpG 5mC identification method, we quantified the CpG methylation levels across different genomic regions, measured by the percentages of methylated CpG sites normalised against the species-specific genomic background. For all species, exonic regions had significantly higher CpG methylation levels than the introns and the genomic background (two-sided binomial test: $p < 10e\text{-}5$). Intergenic regions were significantly depleted of methylated CpG sites (two-sided binomial test: $p < 10e\text{-}5$) (**Supplementary Fig. A**). Also, CpG methylation levels in 3 prime untranslated regions (3' UTRs) were significantly higher than in 5' UTR. These results are consistent with previous findings in *Z. nevadensis* and *B. germanica* genomes [1–3], demonstrating the validity of using PacBio HiFi reads to identify CpG 5mC in termite genomes.

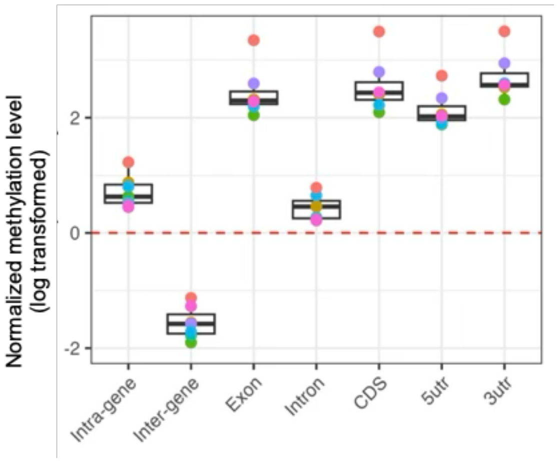

Supplementary Figure A. Boxplots of log transformed normalised CpG site methylation levels in different genomic regions in the seven termite genomes. Normalised methylation level was calculated as the ratio of the percentage of methylated CpG sites in the target genomic region to that in the whole genome. After log transformation, a positive value indicates a higher CpG methylation level than the genomic background and a negative value indicates a lower one. Each colour represents one termite species.

Supplementary Note 3. Associations between TE methylation level and TE abundance at the superfamily level

TE methylation levels can be positively or negatively associated with TE abundance at the superfamily level across the termite phylogeny (**Supplementary Fig. B**). For example, TE abundance in *Pao*, an LTR retrotransposon superfamily, showed a positive association between methylation level and abundance, whereas *Maverick* and *MULE*, two DNA transposon superfamilies, showed negative associations. Interestingly, while *Pao* comprises a mix of recently expanded (low mean Kimura's distance; KD < 20) and decayed families (KD > 20), *MULE* and *Maverick* in Geoisoptera consist mostly of recently expanded families that are hypo-methylated (**Supplementary Fig. C**), consistent with the suppressive role of DNA methylation on TE activity. These results suggest that the direction of the association between TE abundance and methylation at the superfamily level may be shaped by the invasion histories of constituent TE families.

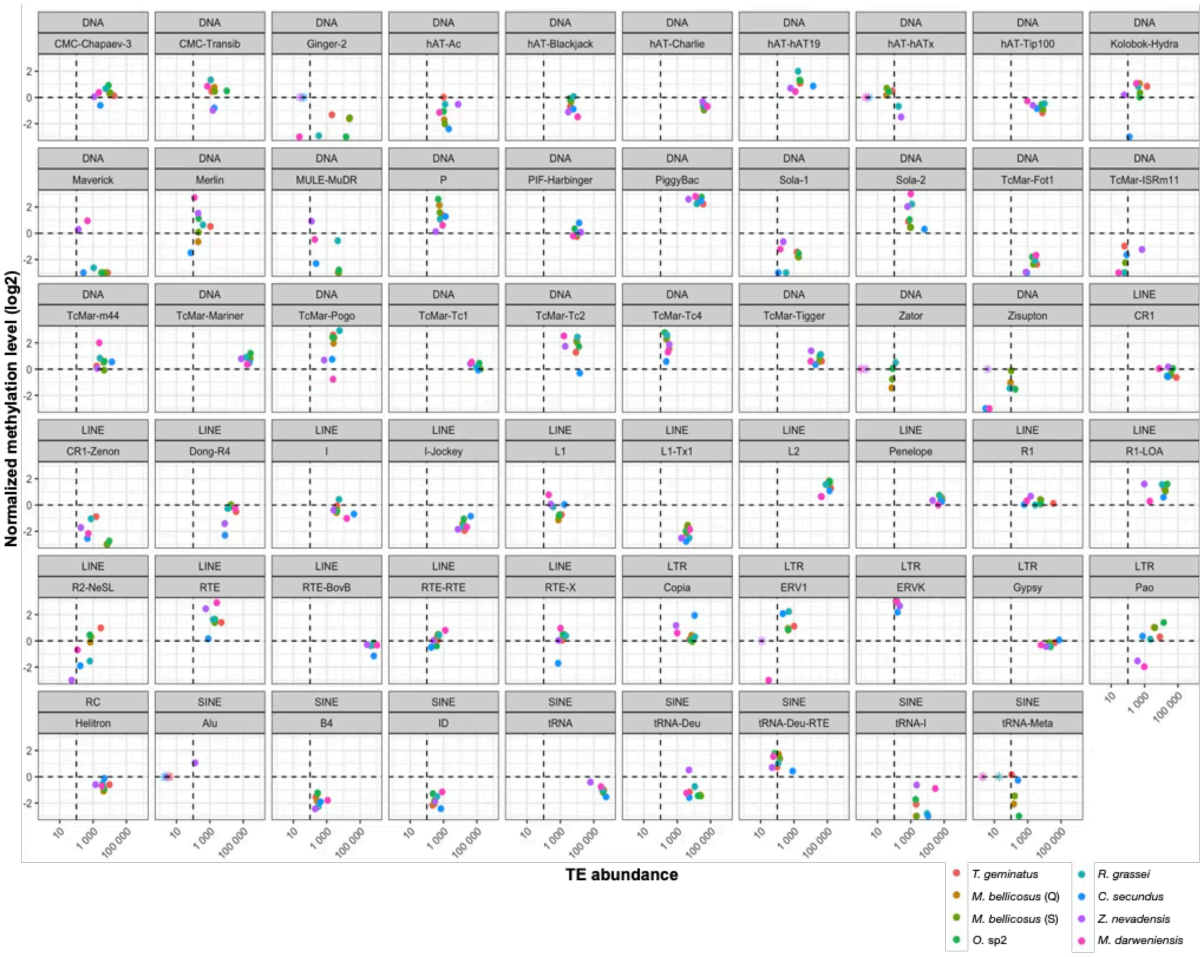

Supplementary Figure B. Association between TE abundance (x-axis) and normalized TE methylation level (y-axis, $\log_2$ scale) for 59 TE superfamilies across the termite phylogeny. For TE superfamilies that were species-specific (e.g., Alu elements in *Z. nevadensis*), normalized DNA methylation values were not available in other species and were thus set to zero; these TE superfamilies are marked with an asterisk (*). The horizontal dashed line represents the normalized genomic methylation level (0) and the vertical dashed line represents 100 TE copies.

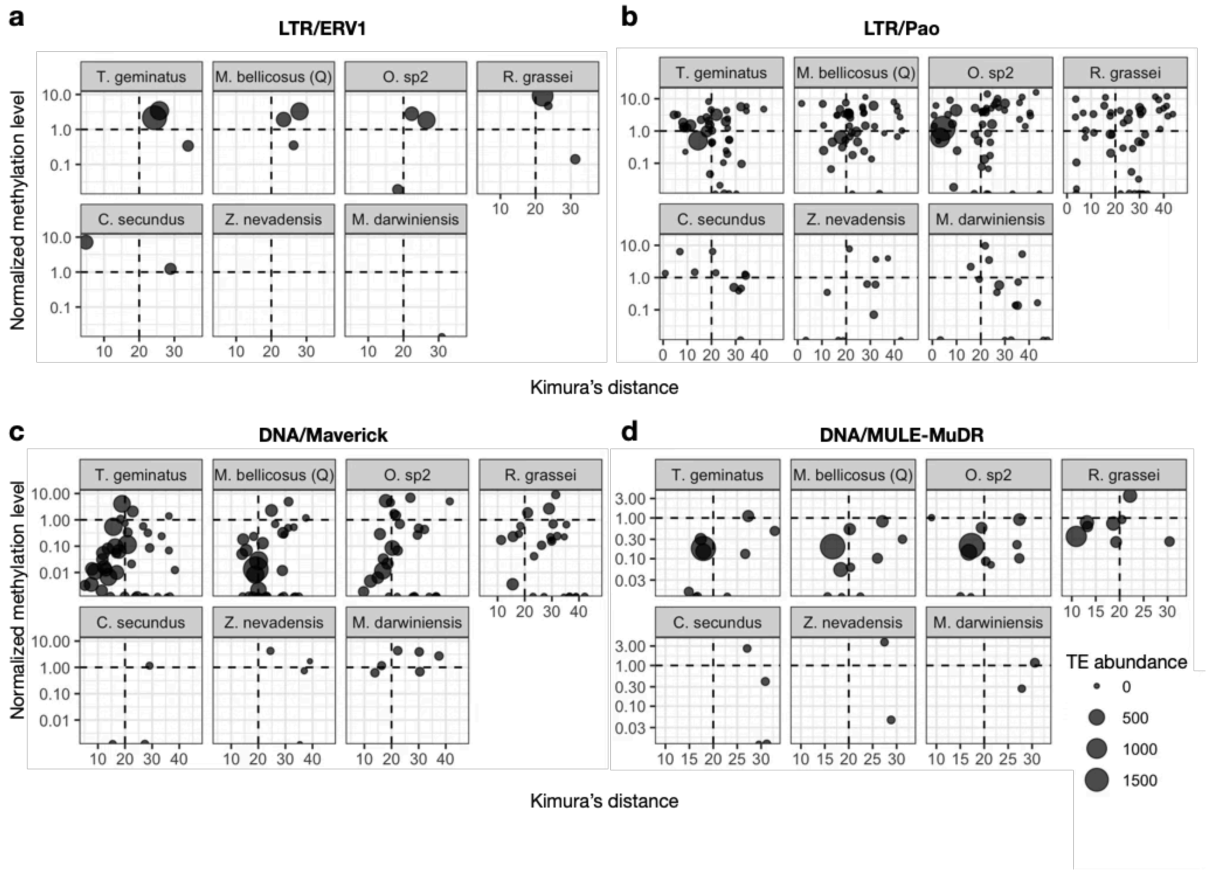

Supplementary Figure C. ERV1 and Pao (panel **a** and **b**), two LTR retrotransposon superfamilies with high DNA methylation levels in species with high TE abundance, consist either of decayed TE families (Kimura's distance, KD > 20) or a mixture of recently expanded (KD < 20) and decayed TE families. In contrast, Maverick and MULE (panel **c** and **d**), two DNA transposon superfamilies with low DNA methylation levels in species with high TE abundance, consist primarily of recently expanded TE families, consistent with the suppressive role of DNA methylation on TE activity. These patterns suggest that the relationship between TE abundance and methylation at the superfamily level may reflect the evolutionary histories of the underlying TE families. Each dot represents one TE family, with the dot size, x value, and y value indicating the abundance, KD, and normalized methylation level of the TE family, respectively.

Supplementary Note 4. Inferring active TE families from TE derived structure variants (SVs)

TE-derived SVs can arise from both active TEs and inactive TEs that have decayed and been lost in some populations. By quantifying the distribution of SV insertions (relative to the reference genome) across five individual termite samples (10 haplotypes), we found that most insertions occurred in only one or two haplotypes (**Supplementary Fig. D**). Because loss of a TE in the reference genome would result in the same "insertion" being detected across multiple haplotypes, the predominance of haplotype-specific insertions suggests that these SVs represent true TE insertions rather than decayed TE sequences missing from the reference genome.

In contrast, for SV deletions it is more difficult to distinguish between true TE excisions (cut-and-paste events) and TE loss, since both can generate haplotype-specific deletions.

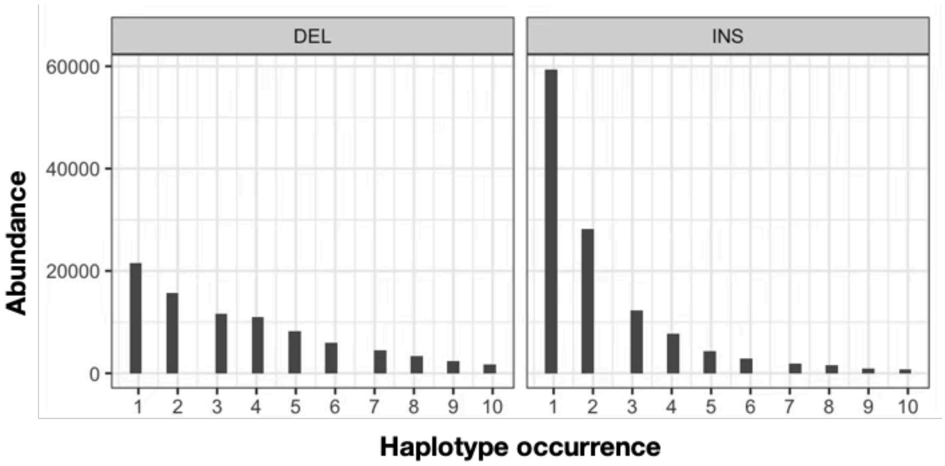

Supplementary Figure D. Haplotype frequency distribution of SV deletion (left) and SV insertion (right) across the five individual termite samples comparing to the reference genome. The majority of SV insertions occur only in one or two haplotypes, indicating these SVs are derived from active TEs.

Supplementary Note 5. Contribution of TE superfamilies to genome size variation among termites

We sorted TE superfamilies by their size variation among termite genomes and found that RTE-BovB (LINE), tRNA (SINE), Gypsy (LTR retrotransposon), TcMar-Mariner (DNA transposon), L2 (LINE), and Tc1/mariner (DNA transposon) show the highest variation. Notably, BovB and Tc1/mariner are also among the top three active TE superfamilies in *M. bellicosus* (together with Ginger-2, a DNA transposon that also exhibits high size variation). This suggests that TE families contribute to genome size variation among termites can still be active.

References

1. Terrapon, N. *et al.* Molecular traces of alternative social organization in a termite genome. *Nat Commun* **5**, 3636 (2014).

2. Glastad, K. M., Gokhale, K., Liebig, J. & Goodisman, M. A. D. The caste- and sex-specific DNA methylome of the termite *Zootermopsis nevadensis*. *Sci. Rep.* **6**, 37110 (2016).

3. Lewis, S. H. *et al.* Widespread conservation and lineage-specific diversification of genome-wide DNA methylation patterns across arthropods. *PLoS Genet.* **16**, e1008864 (2020).

4. Elsner, D., Meusemann, K. & Korb, J. Longevity and transposon defense, the case of termite reproductives. *Proc. Natl. Acad. Sci.* **115**, 5504–5509 (2018).
