# Supplementary material for: Arms races between selfish genetic elements and their host defence in termites
Source: Nat Commun. 2026 Feb 13;17:1702. doi: 10.1038/s41467-026-69550-6 (PMC12909921; doi:10.1038/s41467-026-69550-6)
Supplement: Supplementary file 2 — Description of Additional Supplementary Files [file 41467_2026_69550_MOESM2_ESM.pdf]

Supplementary Data 1. Information and abundance of TE families (a) and superfamilies (b) in termite and wood roach genomes. For each TE family, its consensus sequence length, GC content, classification and mean Kimura distance (within family divergence) is provided.

Supplementary Data 2. Mean CpG methylation level of TE superfamilies in termite genomes.

Supplementary Data 3. A full list of TE defence genes and their presence/absence in termite and woodroach genomes.
