# Supplementary material for: Arms races between selfish genetic elements and their host defence in termites
Source: Nat Commun. 2026 Feb 13;17:1702. doi: 10.1038/s41467-026-69550-6 (PMC12909921; doi:10.1038/s41467-026-69550-6)
Supplement: Supplementary file 6 — Reporting Summary [file 41467_2026_69550_MOESM6_ESM.pdf]

nature portfolio

Corresponding author(s): Bitao Qiu, Judith Korb

Last updated by author(s): Nov 25, 2025

Reporting Summary

Nature Portfolio wishes to improve the reproducibility of the work that we publish. This form provides structure for consistency and transparency in reporting. For further information on Nature Portfolio policies, see our Editorial Policies and the Editorial Policy Checklist.

Statistics

For all statistical analyses, confirm that the following items are present in the figure legend, table legend, main text, or Methods section.

n/a	Confirmed	
☐	☒	The exact sample size (*n*) for each experimental group/condition, given as a discrete number and unit of measurement
☐	☒	A statement on whether measurements were taken from distinct samples or whether the same sample was measured repeatedly
☐	☒	The statistical test(s) used AND whether they are one- or two-sided<br>*Only common tests should be described solely by name; describe more complex techniques in the Methods section.*
☐	☒	A description of all covariates tested
☐	☒	A description of any assumptions or corrections, such as tests of normality and adjustment for multiple comparisons
☐	☒	A full description of the statistical parameters including central tendency (e.g. means) or other basic estimates (e.g. regression coefficient) AND variation (e.g. standard deviation) or associated estimates of uncertainty (e.g. confidence intervals)
☐	☒	For null hypothesis testing, the test statistic (e.g. *F*, *t*, *r*) with confidence intervals, effect sizes, degrees of freedom and *P* value noted<br>*Give P values as exact values whenever suitable.*
☒	☐	For Bayesian analysis, information on the choice of priors and Markov chain Monte Carlo settings
☐	☒	For hierarchical and complex designs, identification of the appropriate level for tests and full reporting of outcomes
☐	☒	Estimates of effect sizes (e.g. Cohen's *d*, Pearson's *r*), indicating how they were calculated

Our web collection on statistics for biologists contains articles on many of the points above.

Software and code

Policy information about availability of computer code

Data collection	No software was used to collect the data.
Data analysis	We have used the following software to analysis the data:<br><br>Genome assembly, genome and TE annotations:<br>Hifiasm (v.0.19.5)<br>BUSCO (v.5.8.2)<br>Merqury (v1.3)<br>GenomeScope 2.0<br>purge_dups (v. 1.2.6)<br>FCS-GX<br>RepeatModeler (v. 2.0.5)<br>MCHelper (v1.6.6)<br>RepeatMasker (v. 4.1.7)<br>Braker3 (v3.0.8)<br>StringTie (v2.2.1)<br>Hisat2 (v. 2.2.1)<br>Transdecoder (v5.71)<br>InterProScan (v5.68)<br>One-code-to-find-them-all

Structure variant annotation:
Sniffles2 pipeline (v2.4)
Minimap (v2.28)
blastn (v2.16)

DNA methylation detection and analysis:
Jasmine (v. 2.0.0)
pbmm2 (v. 1.12.0)
pb-CpG-tools (v.2.3.2)
GenomicRanges

PCA and heatmap:
FactoMineR
pheatmap

Detect positive selection:
Miniprot (v0.13)
MACSE pipeline (v12.01)
HyPhy (v.2.5.62)

The custom shell and R scripts used to analysis the data are available on https://github.com/BitaoQiu/termites_TE_methylation.

For manuscripts utilizing custom algorithms or software that are central to the research but not yet described in published literature, software must be made available to editors and reviewers. We strongly encourage code deposition in a community repository (e.g. GitHub). See the Nature Portfolio guidelines for submitting code & software for further information.

Data

Policy information about availability of data

All manuscripts must include a data availability statement. This statement should provide the following information, where applicable:

- Accession codes, unique identifiers, or web links for publicly available datasets
- A description of any restrictions on data availability
- For clinical datasets or third party data, please ensure that the statement adheres to our policy

The PacBio HiFi reads (including kinetic information used to infer methylation levels) for genome assembly and the Macrotermes bellicosus HiFi reads generated for population genomics have been deposited in the NCBI Sequence Read Archive (SRA) under BioProject accession PRJNA1033592 (https://www.ncbi.nlm.nih.gov/bioproject/PRJNA1033592). The termite genome assemblies, the non-redundant termite repeat library, repeat annotations, repeat abundance data, and summary tables of repeat methylation levels for all studied species are available on Zenodo (https://doi.org/10.5281/zenodo.17704238) or GitHub (https://github.com/BitaoQiu/termites_TE_methylation).

Research involving human participants, their data, or biological material

Policy information about studies with human participants or human data. See also policy information about sex, gender (identity/presentation), and sexual orientation and race, ethnicity and racism.

Reporting on sex and gender	*Use the terms sex (biological attribute) and gender (shaped by social and cultural circumstances) carefully in order to avoid confusing both terms. Indicate if findings apply to only one sex or gender; describe whether sex and gender were considered in study design; whether sex and/or gender was determined based on self-reporting or assigned and methods used. Provide in the source data disaggregated sex and gender data, where this information has been collected, and if consent has been obtained for sharing of individual-level data; provide overall numbers in this Reporting Summary. Please state if this information has not been collected. Report sex- and gender-based analyses where performed, justify reasons for lack of sex- and gender-based analysis.*
Reporting on race, ethnicity, or other socially relevant groupings	*Please specify the socially constructed or socially relevant categorization variable(s) used in your manuscript and explain why they were used. Please note that such variables should not be used as proxies for other socially constructed/relevant variables (for example, race or ethnicity should not be used as a proxy for socioeconomic status). Provide clear definitions of the relevant terms used, how they were provided (by the participants/respondents, the researchers, or third parties), and the method(s) used to classify people into the different categories (e.g. self-report, census or administrative data, social media data, etc.) Please provide details about how you controlled for confounding variables in your analyses.*
Population characteristics	*Describe the covariate-relevant population characteristics of the human research participants (e.g. age, genotypic information, past and current diagnosis and treatment categories). If you filled out the behavioural & social sciences study design questions and have nothing to add here, write "See above."*
Recruitment	*Describe how participants were recruited. Outline any potential self-selection bias or other biases that may be present and how these are likely to impact results.*
Ethics oversight	*Identify the organization(s) that approved the study protocol.*

Note that full information on the approval of the study protocol must also be provided in the manuscript.

Field-specific reporting

Please select the one below that is the best fit for your research. If you are not sure, read the appropriate sections before making your selection.

☐ Life sciences ☐ Behavioural & social sciences ☒ Ecological, evolutionary & environmental sciences

For a reference copy of the document with all sections, see nature.com/documents/nr-reporting-summary-flat.pdf

Ecological, evolutionary & environmental sciences study design

All studies must disclose on these points even when the disclosure is negative.

Study description	We generated individual genomes and DNA methylation profiles from seven termite species (15 individual genomes) with PacBio genome sequencing and examined the co-evolution patterns between transposable elements and DNA methylation during termite evolution.
Research sample	One queen, one soldier and three workers from five different Macrotermes bellicosus colonies, one Odontotermes sp.2 queen, one Reticulitermes grassei queen, one Cryptotermes secundus worker, one Mastotermes darwiniensis worker, one Zootermopsis nevadensis worker, one Trinervitermes geminatus worker were collected for the individual genome sequencing.
Sampling strategy	We collected one individual from each species. To ensure reproducibility, we included both a queen and a soldier from two different Macrotermes bellicosus colonies. Principal component analysis (PCA) and clustering analyses showed that these two samples were more similar to each other than to samples from other species. Furthermore, the strong phylogenetic signal observed supports the use of individual samples as reliable representatives of their respective species.
Data collection	For Cryptotermes secundus, Mastotermes darwiniensis, Reticulitermes grassei and Zootermopsis nevadensis, a queen (for R. grassei) or a worker (for other species) had been collected from lab colonies housed at the University of Freiburg. C. secundus had been collected at Channel Island, Australia, in 2019; M. darwiniensis colony in Darwin, Australia, in 2022, R. grassei on Île d'Oléron, France, in 2020 (donated by Franck Dedeine), Z. nevadensis in 2023 in California, the United States. These colonies had been kept in climate chambers in Freiburg, Germany, and the individual termites were freshly killed in 2023 to extract DNA.<br><br>For the other species, samples had been collected in the field and stored in 100% ethanol or RNAlater at -20 °C until DNA extraction. A Trinervitermes geminatus worker in Lamto, Côte d'Ivoire, in 2021; a Odontotermes sp.2 queen in the Comoé National Park, Côte d'Ivoire, in 2019; a Macrotermes bellicosus queen and a minor soldier in the Comoé National Park, Côte d'Ivoire in 2017. Besides, four minor workers of M. bellicosus were collected from different colonies between 2018 and 2019 in the Comoé National Park, Côte d'Ivoire to detect structure variants (SVs) in M. bellicosus.
Timing and spatial scale	Termite samples were collected from 2019 to 2023. See Data collection.
Data exclusions	No data were excluded.
Reproducibility	We have employed different TE annotation pipelines, structure variant pipelines, and thresholds for quantifying active TEs and annotation TE ages. The results are highly consistent, supporting reproducibility of our study.
Randomization	Individual samples were initially collected at random from field sites or laboratory colonies. For each species, we collected multiple individuals (preferably larger ones to ensure sufficient DNA yield) and extracted genomic DNA. We then assessed DNA quality (fragment length, yield, and Nanodrop metrics) and selected the highest-quality sample as the representative for genome sequencing. Thus, although the final sequenced sample was chosen based on DNA quality, the initial sampling procedure was largely random.
Blinding	Not relevant as an exact identification of species is necessary for our comparative analyses.

Did the study involve field work? ☒ Yes ☐ No

Field work, collection and transport

Field conditions	For the collection fieldwork in Lamto and Comoé National Park (Côte d'Ivoire), the temperatures were between 25 to 30 ° C and there were no rains.<br><br>For the collection in Darwin, Australia, temperatures were between 29 to 35 ° C and there were no rains (end of dry season). Because live termites (in woodblocks) were transported to Freiburg, Germany, the field condition in Australia is not relevant.
Location	Lamto and Comoé National Park in Côte d'Ivoire. Gunn Point in Australia.
Access & import/export	For the collection in Côte d'Ivoire, the collection and export permits were provided by B. Sinsin and the Office Ivoirien des Parcs et Réserves.

	For the collection in Darwin, Australia, the collection (permit number 64452 & 71896) and exporting (permit PWS2019-AU-000897) permits were provided by the Parks and Wildlife Commission, Northern Territory, the Department of the Environment, Water, Heritage and the Arts.
Disturbance	No disturbance

Reporting for specific materials, systems and methods

We require information from authors about some types of materials, experimental systems and methods used in many studies. Here, indicate whether each material, system or method listed is relevant to your study. If you are not sure if a list item applies to your research, read the appropriate section before selecting a response.

Materials & experimental systems

n/a	Involved in the study
☒	☐ Antibodies
☒	☐ Eukaryotic cell lines
☒	☐ Palaeontology and archaeology
☐	☒ Animals and other organisms
☒	☐ Clinical data
☒	☐ Dual use research of concern
☒	☐ Plants

Methods

n/a	Involved in the study
☒	☐ ChIP-seq
☒	☐ Flow cytometry
☒	☐ MRI-based neuroimaging

Animals and other research organisms

Policy information about studies involving animals; ARRIVE guidelines recommended for reporting animal research, and Sex and Gender in Research

Laboratory animals	Cryptotermes secundus (a worker individual, sex unknown, 3-4 year old, large size), Mastotermes darwiniensis (a worker individual, sex unknown, 1-2 year old, large size), Reticulitermes grassei (a queen individual , female, > 3 year old) and Zootermopsis nevadensis (a worker individual, sex unknown, large size, 1-2 year old).
Wild animals	Macrotermes bellicosus: Queen (female, >7 years old), collected in Comoé National Park, Côte d'Ivoire; preserved in RNAlater prior to sequencing. Minor soldier (male, a few months old), collected in Pendjari National Park, Benin; preserved in 100% ethanol prior to sequencing. Three major workers (male, young <1 year), collected in Comoé National Park, Côte d'Ivoire; preserved in 100% ethanol prior to sequencing.<br>Odontotermes sp. 2: Queen (female, ~4 years old), collected in the field in Comoé National Park, Côte d'Ivoire; preserved in RNAlater prior to sequencing.<br>Trinervitermes geminatus: Worker (sex unknown), collected in the field in Lamto, Côte d'Ivoire; preserved in 100% ethanol prior to sequencing.<br><br>All individuals were captured with forceps and killed with Scalpel knife in the field for subsequent DNA extraction. Samples were preserved in RNAlater or 100% ethanol, transported to Freiburg (Germany) by air, and stored at –20 °C until DNA extraction.
Reporting on sex	Queen individuals are females. For workers samples, sex are unknown because termite workers are immature (without developed genital). Minor soldier of Macrotermes bellicosus, male.
Field-collected samples	Cryptotermes secundus, Reticulitermes grassei, Mastotermes darwiniensis, Zootermopsis nevadensis colonies had been kept in climate chambers in Freiburg, Germany, and the individual termites were freshly killed in 2023 to extract DNA.
Ethics oversight	No ethical approval is needed because termites are insects and they are not endanger animals.

Note that full information on the approval of the study protocol must also be provided in the manuscript.

Plants

Seed stocks	*Report on the source of all seed stocks or other plant material used. If applicable, state the seed stock centre and catalogue number. If plant specimens were collected from the field, describe the collection location, date and sampling procedures.*
Novel plant genotypes	*Describe the methods by which all novel plant genotypes were produced. This includes those generated by transgenic approaches, gene editing, chemical/radiation-based mutagenesis and hybridization. For transgenic lines, describe the transformation method, the number of independent lines analyzed and the generation upon which experiments were performed. For gene-edited lines, describe the editor used, the endogenous sequence targeted for editing, the targeting guide RNA sequence (if applicable) and how the editor was applied.*
Authentication	*Describe any authentication procedures for each seed stock used or novel genotype generated. Describe any experiments used to assess the effect of a mutation and, where applicable, how potential secondary effects (e.g. second site T-DNA insertions, mosiacism, off-target gene editing) were examined.*
